# Guard-cell phytochromes impact seedling photomorphogenesis and rosette leaf morphology

**DOI:** 10.17912/micropub.biology.000521

**Published:** 2022-01-31

**Authors:** Sookyung Oh, Que Kong, Beronda L. Montgomery

**Affiliations:** 1 DOE-Plant Research Laboratory, Michigan State University, East Lansing, MI 48824, USA; 2 Department of Biochemistry and Molecular Biology, Michigan State University, East Lansing, MI 48824, USA; 3 Department of Microbiology & Molecular Genetics, Michigan State University, East Lansing, MI 48824, USA

## Abstract

Using a previously established transgenic approach to inactivate phytochrome chromophore synthesis in specific organs or tissues, we used a guard cell-specific promoter to induce phytochrome deficiencies in guard cells of *Arabidopsis thaliana*. Analyses of multiple homozygous lines depleted of phytochromes in stomatal guard cells indicated elongated hypocotyls specifically in red and far-red growth conditions. Furthermore, rosette leaves of adult plants with guard cell-specific phytochrome deficiencies showed enhanced serration compared to the wild-type Col-0 parent. Thus, we demonstrate that guard cell-localized phytochromes impact the inhibition of hypocotyl elongation, as well as leaf margin morphology of adult rosette leaves in *A. thaliana*.

**Figure 1. Phenotyping of tissue-specific biliverdin reductase (BVR) expressing lines:**
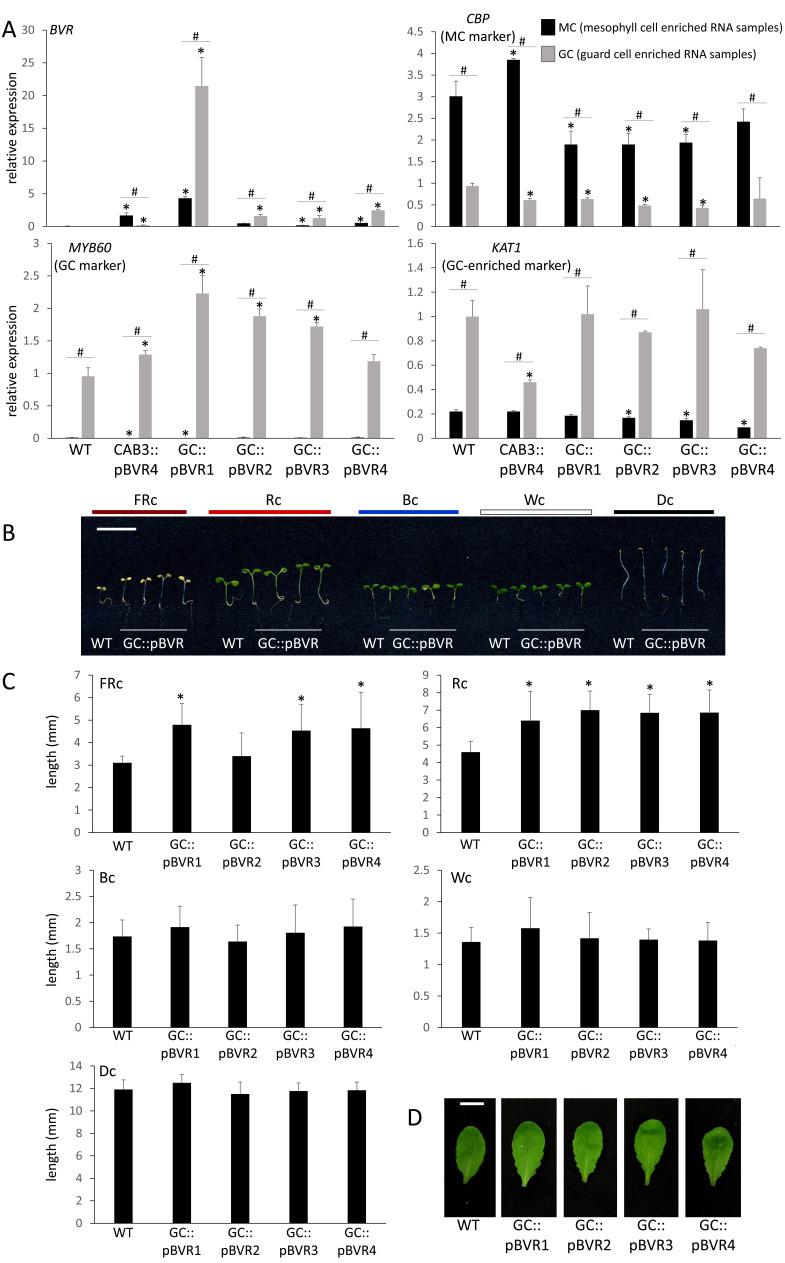
A) Tissue-specific marker gene expression in BVR lines. Expression of *BVR* (encoding BVR, UniProt: P46844), mesophyll cell (MC) marker gene *CALMODULIN-BINDING PROTEIN* (*CBP; At4g33050*; Cho *et al.*, 2012), guard cell (GC) marker gene *MYB DOMAIN PROTEIN 60* (*MYB60; At1g08810*; Cominelli *et al.*, 2005), and GC marker gene *POTASSIUM (K+) CHANNEL IN ARABIDOPSIS THALIANA 1* (*KAT1; At5g46240*; Pilot *et al.*, 2001) measured via qRT-PCR from RNA from mesophyll cell (MC) or guard-cell epidermal enrichment peel (GCEEP) enrichments from ~ 5-week old wild-type (WT) and GC::pBVR plants with GC-localized, plastid-targeted *BVR* (pBVR) expression grown in short-day conditions at 100 µmol m^-2^ s^-1^. Unpaired, two-tailed Student’s t test comparing expression to Col-0 WT for either MC or GCEEP fraction, *, p<0.05 and comparing expression for MC and GCEEP for WT or each BVR line, #, p<0.05. (B) Images of representative seedlings for Col-0 WT and four independent GC::pBVR lines. Seedlings were grown on MS medium for 7 d under constant light of far-red (FRc; 5 μmol m^-2^ s^-1^), red (Rc; 50 μmol m^-2^ s^-1^), blue (Bc; 25 μmol m^-2^ s^-1^), white (Wc; 100 μmol m^-2^ s^-1^), or darkness (Dc). (C) Mean hypocotyl lengths of seedlings (±SD, n≥15) under FRc, Rc, Bc, Wc, and Dc conditions. Unpaired, two-tailed Student’s t test comparing transgenic lines to Col-0 WT, *, p<0.05. (D) Adult vegetative leaf morphology phenotypes for Col-0 WT and GC::pBVR lines. Plants were grown on soil in 100 μmol m^-2^ s^-1^ W light under long days (16 h light, 8 h dark) for 21 days and fully expanded fifth rosette leaves are shown. Bar, 1 cm.

## Description

Light impacts growth and development throughout the plant life cycle; however, photomorphogenesis occurs differently in individual tissues and organs (Montgomery, 2016). Light actively inhibits elongation in the hypocotyl, but promotes the growth and development of cotyledons, leaves, and roots. Distinct responses in specific tissues are maintained to some extent through the differential accumulation of photoreceptors such as phytochromes in distinct tissues and at different times during development (Adam *et al.*, 1994; Somers and Quail, 1995a, 1995b; Goosey *et al.*, 1997; Nagatani, 1997; Tóth *et al.*, 2001; Sharrock and Clack, 2002; Baba-Kasai *et al.*, 2014; van Gelderen *et al.*, 2018); as well as in large part due to distinct signal transduction pathways downstream of photoreceptors in distinct tissues and organs (Bou-Torrent *et al.*, 2008; Montgomery, 2008; Endo *et al.*, 2016; Montgomery, 2016).

Stomata are specialized epidermal cells that occur in the epidermis for all above-ground tissues and serve to facilitate gas exchange and water uptake central to photosynthesis. Stomata respond to environmental signals, including light (Roth-Bejerano and Itai, 1981; Kinoshita *et al.*, 2001; Talbott *et al.*, 2003; Boccalandro *et al.*, 2009; Casson *et al.*, 2009; Matthews *et al.*, 2020; Zhu *et al.*, 2020). Specific phytochromes have been shown to impact stomatal development and function, with phyB promoting the stomatal index, or the percentage of stomata cells out of the total number of epidermal cells, and regulation of stomatal opening in red light (Boccalandro *et al.*, 2009; Casson *et al.*, 2009; Kang *et al.*, 2009; Wang *et al.*, 2010; Casson and Hetherington, 2014). The photoreceptor phyA stimulates production of stomata in far-red light (Kang *et al.*, 2009). Notably, phytochromes are localized in guard cells themselves (Somers and Quail, 1995b; Kang *et al.*, 2009), and thus stomatal responses can potentially be controlled by photoreceptors locally or through intercellular communication.

Tissue-specific expression of *PHY* genes in *phy* mutant backgrounds has been used to explore spatial-specific roles of phytochromes. In regards to the function of phyB in regulating stomata, recent analyses used tissue-specific *PHYB* (*At2g18790*) expression to explore phytochrome-dependent roles in guard cells (Casson and Hetherington, 2014). Expression of *PHYB* in guard cells demonstrated that phyB accumulation in these cells was sufficient to rescue a defect in photoregulation of the stomatal index in a *phyB* mutant (Casson and Hetherington, 2014). These plant lines with guard cell-specific *PHYB* expression also exhibited larger leaves, potentially indicating more extensive growth-related impacts of guard cell-specific phytochrome function. Prior investigations also correlated stomatal regulation with control of additional whole plant growth phenotypes, including correlating the regulation of stomatal opening with vegetative development in terms of the hypocotyl length of seedlings (Xinhong *et al.*, 2011), and the rosette and flowering time at the adult stage (Kinoshita *et al.*, 2011; Ando *et al.*, 2013).

To investigate the function of phytochromes in guard cells, we used a previously verified transgenic approach for expressing a phytochrome chromophore-inactivating enzyme, biliverdin reductase (BVR; UniProt: P46844) to regulate accumulation of photoactive phytochromes in planta (Lagarias *et al.*, 1997; Montgomery *et al.*, 1999; Montgomery *et al.*, 2001; Warnasooriya and Montgomery, 2009; Costigan *et al.*, 2011). We used the promoter p*GC1* (*At1g22690*), which drives high-level, guard-cell specific gene expression and has low expression activity in mesophyll cells (Yang *et al.*, 2008), to generate plant lines with guard cell-localized, plastid-targeted BVR (pBVR) accumulation to induce guard cell-specific phytochrome depletion. We used these lines to examine the roles of guard cell-localized phytochromes in distinct aspects of photomorphogenesis.

We successfully isolated four lines with guard-cell enriched expression of BVR, i.e., GC::pBVR1 to GC::pBVR4 as verified by qRT-PCR analyses with RNA extracted from mesophyll cell (MC) and guard cell enriched epidermal peel (GCEEP) fractions and compared to MC and GCEEP fractions from the CAB3::pBVR representative line with MC-localized *BVR* expression (Fig 1A; Warnasooriya and Montgomery, 2009). We observed significant enrichment of *BVR* expression in GCEEP compared to MC in several GC::pBVR lines, confirming guard-cell enriched *BVR* expression driven by the GC promoter. To confirm the reliability of our MC and GCEEP fractions, we tested RNA fractions for expression of an MC-enriched marker gene, i.e., *CALMODULIN-BINDING PROTEIN* (*CBP; At4g33050*) (Cho *et al.*, 2012), and guard-cell marker genes, i.e., *POTASSIUM (K+) CHANNEL IN ARABIDOPSIS THALIANA 1* (*KAT1; At5g46240*) (Pilot *et al.*, 2001) and *MYB DOMAIN PROTEIN 60* (*MYB60; At1g08810*) (Cominelli *et al.*, 2005). As expected, we detected preferential expression of *CBP* in the MC RNA enrichment and of *KAT1* and *MYB60* in the GCEEP RNA enrichment (Fig 1A). Additionally, we assessed protein extracts from GCEEP for BVR enzymatic activity using a previously described specific activity assay (Lagarias *et al.*, 1997). Whereas no BVR activity was detected for GCEEP protein extracts from WT and a CAB3::pBVR line, we detected BVR activity in the range of 0.2 – 2.8 (I.U. mg^−1^) × 10^3^ for GCEEP extracts from GC::pBVR lines.

We assessed light-dependent growth of GC::pBVR seedlings compared to Col-0 WT seedlings under constant blue (Bc), red (Rc), far-red (FRc) and white (Wc) light. We observed significantly elongated GC::pBVR seedlings under both FRc and Rc illumination (Fig 1B and 1C). GC::pBVR seedlings were not significantly different relative to WT seedlings in the dark or under either Bc or Wc light (Fig 1B and 1C), which is distinct from phenotypes observed due to depletion of phytochromes in mesophyll cells as CAB3::pBVR lines were previously reported to have elongated hypocotyls in Wc, FRc, Rc, and Bc (Warnasooriya and Montgomery, 2009; Warnasooriya *et al.*, 2011). The disruption in light-dependent inhibition of hypocotyl elongation only under R and FR light for GC::pBVR lines is notable as these are wavelengths specifically associated with phytochrome-regulated functions. Although not previously associated specifically with light-dependent regulation, prior analyses of plants with altered ABA-regulated stomatal function showed a correlation between higher rates of stomatal closure and elongated hypocotyls (Xinhong *et al.*, 2011).

For adult plants, GC::pBVR plants exhibited altered leaf shape (i.e., enhanced serration) under long-day conditions of W light at 100 µmol m^-2^ s^-1^ (Fig 1D). In Arabidopsis, leaf heteroblasty can be indicated by leaf margin serration. As the major disruption at the adult leaf stage for GC::pBVR lines was an alteration in leaf serration or leaf margin morphology defects, these plants may exhibit alterations in heteroblasty correlated with guard-cell phytochrome deficiency. Relatedly, prior analyses with tissue-specific expression of *phyB* also indicated a correlation between a role for guard cell-localized phyB and leaf phenotypes. The leaves of *phyB* mutants are significantly different from WT with less leaf area and elongated petioles (Casson and Hetherington, 2014). When a *phyB* mutant was specifically complemented with a guard cell-localized *PHYB* construct, i.e., SPCH::PHYB, the leaf phenotype was largely complemented, in addition to the stomatal index phenotype (Casson and Hetherington, 2014). Together, these associations of the regulation of leaf phenotypes with stomatal phytochrome pools indicate key roles of GC-localized phytochromes in regulating whole leaf phenotypes.

## Methods

**Plasmid construction**

We constructed plant transformation vector pORE_O3_GC/TPBVR using a TP-*BVR* fragment for plastid-targeted BVR protein accumulation from the previously described CAB3::pBVR construct pBIB/CAB3-TPBVR (Warnasooriya and Montgomery, 2009) and guard cell-specific *GC* promoter (Yang *et al.*, 2008). Specifically, the guard-cell specific, plastid-targeted *BVR* construct (GC::pBVR) was made by isolating the transit peptide-BVR (pBVR) fragment from pBIB/CAB3-TPBVR using *Xba*I and *Sac*I and subcloning into *Xba*I and *Sac*I digested pORE_O3_GC plasmid, which contains the *GC1* promoter [p*GC*1 (*At1g22690*)], by ligation to create the pORE_O3_GC/TPBVR plasmid.

**Plant transformation and growth conditions**

*Arabidopsis thaliana* (Columbia or Col-0 ecotype) plants were used as wild-type (WT) and for transformation of all constructs. We used both pBIB/CAB3-TPBVR and pORE_O3_GC/TPBVR constructs in *Agrobacterium tumefaciens* strain GV3101. We isolated multiple single insertion homozygous lines that were screened for *BVR* expression, including enrichment in the mesophyll or guard cells for the CAB3::pBVR4 (also denoted as CAB3::pBVR as the sole line used in these analyses) and GC::pBVR lines, respectively.

For hypocotyl growth analyses, surface sterilized seeds were plated and grown on MS medium containing 1% (w/v) sucrose and 0.7% (w/v) Phytoblend agar (Caisson Labs, UT) at 22°C for 7 d under the indicated light conditions. Light sources used included blue (B; λmax ∼470 nm), far-red (FR; λmax ∼735 nm), red (R; λmax ∼670 nm), and white (W) as previously described (Warnasooriya and Montgomery, 2009). Light intensities were measured using a LI-COR LI-250A Light Meter with inline LI-COR quantum sensor for R, B and W light, and using a StellarNet EPP2000 spectroradiometer (Apogee Instruments) for FR light.

**Phenotyping**

We determined light-dependent hypocotyl elongation of seedlings under specified growth conditions by scanning images of seedlings and quantifying hypocotyl length using ImageJ software as previously described (Warnasooriya and Montgomery, 2009). For phenotypic observation of rosette leaf morphology, plants were grown in a long-day growth chamber with 16 h/8 h day/night light cycle at a white light intensity of ~100 µmol m^-2^ s^-1^ and 22°C/20°C day/night temperature cycles.

**Gene expression analysis**

RNA samples were extracted using the RNeasy® Plant Minikit (Qiagen, CA) as previously described (Oh and Montgomery, 2013) from whole plants, mesophyll cells from ~15 g of 5 to 6 week-old plant leaves (Yoo *et al.*, 2007) or guard cell-enriched epidermal peels from adult plants grown in a short-day growth chamber with 8 h/16 h day/night light cycles at a white light intensity of 100 µmol m^-2^ s^-1^ and 22°C/20°C day/night temperature cycle, according to the protocol of (Zhu *et al.*, 2016). Quantitative RT-PCR (qRT-PCR) was performed essentially as described (Oh and Montgomery, 2013). Expression of *BVR* and tissue-specific marker genes was assessed, including mesophyll cell-enriched marker *CBP* (*At4g33050*)and guard cell marker genes *KAT1* (*At5g46240*) and *MYB60* (*At1g08810*). The control genes used for qRT-PCR analyses were *PP2A* (*At1g13320*) and *ACT2* (*At3g18780*). Primers for each gene are listed in Table 1.

## Reagents

**Table 1**. Primers for quantitative reverse transcriptase PCR (qRT-PCR)

**Table:** 

	**Forward primer (5ˈ−>3ˈ)**	**Reverse primer (5ˈ−>3ˈ)**
*BVR*	ACAAGGGTCTGCTGTCATGG	GGGACCCAGAAGTGAACTGG
*CBP* (MC marker)	TGTGTTTGATACCCACACAAGGG	ACCCAACTTCAAGACCCATGACC
*MYB60* (GC marker)	ACACTGGGTTATTGAGATGCAGCA	TGGACGCCCATTTGTTACCCAA
*KAT1* (GC marker)	GACGCTGAGTATTTCCCACCAA	GAAGTCCACTGCTCCTGACA
*PP2A* (internal control)	TAACGTGGCCAAAATGATGC	GTTCTCCACAACCGCTTGGT
*ACT2* (internal control)	AGCACCCTGTTCTTCTTACCG	CCAGAATCCAGCACAATACCGG
